# Domestic Sewage Treatment Using a One-Stage ANAMMOX Process

**DOI:** 10.3390/ijerph17093284

**Published:** 2020-05-08

**Authors:** Yuan Wei, Yue Jin, Wenjie Zhang

**Affiliations:** 1 Guangxi Key Laboratory of Environmental Pollution Control Theory and Technology, College of Environmental Science and Engineering, Guilin University of Technology, Guilin 541004, China; wy0000000000@126.com; 2 College of Civil Engineering and Architecture, Guilin University of Technology, Guilin 541004, China; 3 Guangxi Collaborative Innovation Center for Water Pollution Control and Water Safety in Karst Area, Guilin University of Technology, Guilin 541004, China; 2010053@glut.edu.cn

**Keywords:** domestic sewage treatment, ANAMMOX, new process

## Abstract

A one-stage anaerobic ammonium oxidation (ANAMMOX) reactor can be quickly started within 40 days by mixing partial nitrifying sludge with ANAMMOX granular sludge with an average temperature of 30 °C. After 70 days of nitrogen load acclimation, *Acinetobacter,* including *Candidatus Kuenenia,* became the dominant strain of the system within the reactor, which exhibited high efficiency and a stable nitrogen removal performance. At an influent chemical oxygen demand (COD), NH_4_^+^-N content, total nitrogen (TN) content, hydraulic retention time (HRT), temperature, and reactor dissolved oxygen (DO) content of 100, 60, and 70 mg/L, 6 h, 30 ± 1 °C, and below 0.6 mg/L, respectively, the one-stage ANAMMOX reactor could effectively treat domestic sewage on campus. The removal rates of COD, NH_4_^+^-N, and TN were approximately 89%, 96.7%, and 70%, respectively.

## 1. Introduction

With the rapid urbanization in China, the total amount of urban domestic sewage has been increasing. According to data from the National Bureau of Statistics of China, the daily treatment capacity of urban sewage in 2018 was 181,450,000 m^3^, an increase of 19.97% from that in 2014 (151,240,000 m^3^) [1]. The most common wastewater treatment processes used in domestic sewage treatment plants in China include the anaerobic/anoxic/oxic (A^2^/O), anoxic/oxic (A/O), and oxidation ditch processes. However, regardless of the processes adopted, biological nitrogen removal is achieved through aerobic nitrification and anoxic denitrification. In the aerobic stage, sufficient oxygen is required for NH_4_^+^-N nitrification, resulting in high energy consumption [2]. Further, in the anoxic stage, an adequate organic carbon source is needed to meet the denitrification demand [3]. With the continuous societal development and progress, China’s focus on energy conservation and environmental protection is increasing, and the limitations of traditional nitrification/denitrification processes are becoming increasingly clear [4]. Therefore, it is particularly urgent for China, with a population of 1.4 billion, to develop a new, efficient, and energy-saving domestic sewage treatment process.

Since its development in the Kluyver Laboratory of Biotechnology (Department of Microbiology and Enzymology, Delft University of Technology, the Netherlands) in 1995, ANAMMOX has been a focus of research in the field of water treatment [5]. It involves the conversion of NH_4_^+^-N and NO_2_^−^-N into N_2_ by ANNAMOX bacteria under anaerobic or anoxic conditions [6,7,8]. As a new type of wastewater nitrogen removal process [9,10], ANAMMOX has high nitrogen removal efficiency, requires no additional organic carbon source [11], has a high load, low excess sludge output and low operating costs [8,12], and clear advantages over traditional nitrification/ denitrification technologies [13]. Researchers have continuously studied ANAMMOX to determine its potential engineering applications. To date, ANAMMOX has been successfully applied to treat high-ammonia wastewater [14], such as landfill leachate [15], monosodium glutamate production wastewater [16], and pig breeding wastewater [17]. Currently, over 100 ANAMMOX projects are in operation worldwide [18], and the number is continuously increasing. Thus, the application prospects of ANAMMOX are quite broad. However, there are very few engineering applications of ANAMMOX in domestic sewage treatment, and only experimental research has been carried out in laboratories or on a pilot-scale. For example, Li et al. [19] employed ANAMMOX technology to study the start-up and nitrogen removal performance of an oxidation ditch on a pilot scale, in which the average total nitrogen (TN) removal rate reached 82.17%.

The main factors that have hindered the application and development of ANAMMOX processes in domestic sewage treatment include (1) an insufficient supply of NO_2_^−^-N. Strous et al. [6] conducted a quantitative study on the growth physiology of ANAMMOX bacteria in a sequencing batch reactor. The ratio of NO_2_^−^-N/NH_4_^+^-N consumed in the ANAMMOX process was 1.32; thus, an adequate amount of NO_2_^−^-N must be present during the treatment process. (2) The temperature of domestic sewage in some areas is relatively low. According to the results obtained by Kazuichi et al. [20], who studied an anaerobic biofilter (ABF) reactor, the optimal growth temperature range of ANAMMOX bacteria is 30–37 °C; the activity of ANAMMOX bacteria in low-temperature environments is low, which affects the stability of the ANAMMOX process. (3) ANAMMOX bacteria have strict growth environment requirements. They are easily out-grown by other fast-growing bacteria, resulting in a long cycle (up to 32 days) and low cell yield and rendering it difficult for the ANAMMOX process to commence [21,22].

To address these problems, a set of one-stage ANAMMOX reactors with external aeration was designed in this study. Compared with the other one-stage ANAMMOX process, the reactor used here can avoid the direct impact of dissolved oxygen (DO) on ANAMMOX bacteria. The wastewater from the campus of the Guilin University of Technology (Guilin, Guangxi, China) was used as the experimental water. The reactor was operated with partial nitrifying [23] and ANAMMOX granular sludge [24,25] that had been domesticated and cultivated in the laboratory. The one-stage ANAMMOX has a simpler structure, easier operation, and lower sewage treatment cost than those of traditional domestic sewage treatment processes, and is suitable for use in tropical and some subtropical regions with warmer climates. Experiments were conducted in areas of southern China where the temperature is more suitable (the temperature is maintained at 30 °C). The treatment effect of the one-stage ANAMMOX reactor on the chemical oxygen demand (COD), NH_4_^+^-N, and TN was studied in a location with an appropriate temperature (southern China). Furthermore, by conducting high-throughput sequencing and other methods of analyzing the changes in the microbial population of the reactor, the key factors, and best operating parameters that affect the treatment ability of the reactor are determined, and theoretical support for the application of ANAMMOX technology in domestic sewage treatment is provided.

## 2. Materials and Methods

### 2.1. Test Device and Method

**Figure 1:**
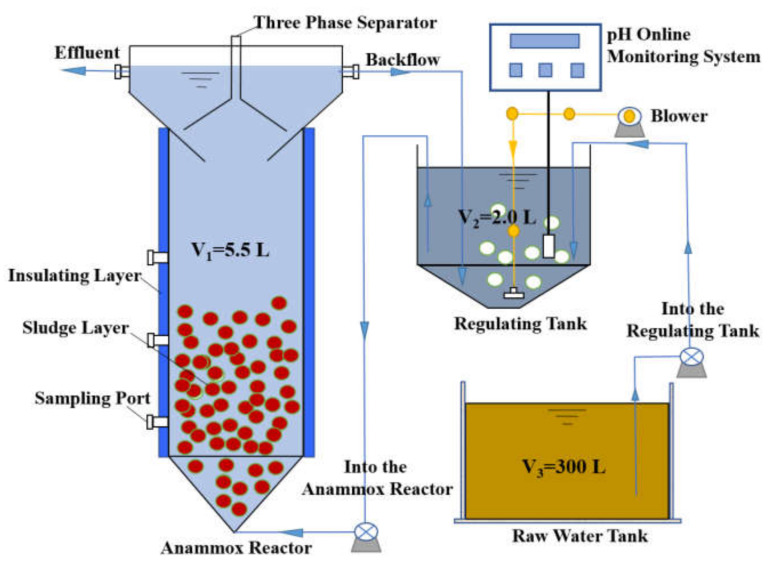
Schematic diagram of the reactor.

In the experiment, an up-flow ANAMMOX reactor was used to treat wastewater. A schematic diagram of the device is presented in Figure 1, which mainly includes the main reactor (effective volume, V_1_ = 5.5 L), regulating tank (effective volume, V_2_ = 2.0 L), and raw water tank (effective volume, V_3_ = 300 L). During the operation of the reactor, wastewater from the raw water tank enters the regulating tank through the water inlet pump to adjust the pH and pre-aeration. Wastewater containing a certain amount of oxygen enters the main reactor to undergo partial nitrification and ANAMMOX reaction, which degrades pollutants in the wastewater. After separation by the three-phase separator at the top of the reactor, one part of the water overflows from the water outlet, and the other part flows back to the regulating tank through the reflux pump. Setting the return water can accelerate the rising speed of the wastewater in the main reactor so that the microorganisms in the upper layer of the reactor can also effectively remove the pollutants.

The experiment was divided into three stages. Artificial wastewater was used in the first two stages, and domestic wastewater from Guilin University of Technology was used in the third stage [26]. The composition of the wastewater used in the three experimental stages is shown in Table 1. During the experiment, the pH was adjusted using 0.4 mol/L H_2_SO_4_ and 0.5 mol/L NaHCO_3_/Na_2_CO_3_. The NH_4_^+^-N in the artificial wastewater was provided by NH_4_HCO_3_, and specific amounts of KH_2_PO_4_, CaCl_2_·2H_2_O, and MgSO_4_·7H_2_O were added to provide microelements for the microorganisms. During the experiment, the reactor temperature was set at 30 ± 1.0 °C, which is reached throughout the year in parts of southern China.

**Figure 2:**
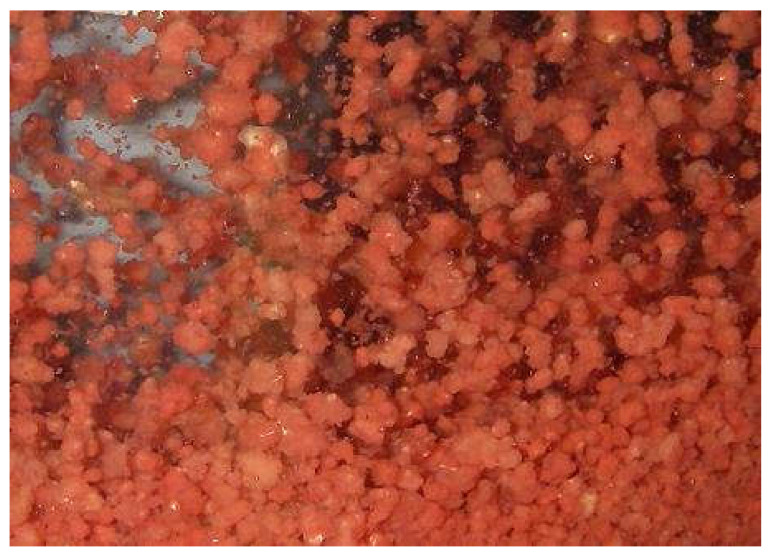
ANAMMOX granular sludge sample.

The partial nitrifying [23] and ANAMMOX [24,25] sludges used in this study were obtained from the sewage treatment station of Guilin University of Technology. ANAMMOX sludge is in the form of red granules and has good activity [27]. Its morphology is shown in Figure 2. The inoculation amounts of the partial nitrifying and ANAMMOX sludges were 1500 and 300 mL, respectively, and the concentrations were 2000 and 3000 mg/L. Bamboo charcoal was used as the microbial carrier in the reactor, which had a filling capacity of 2 L.

The operation conditions of each stage are shown in Table 2. The start-up period lasted for 37 days, and its main purpose was to adapt the inoculated partial nitrifying and ANAMMOX sludge to the culture environment and operating conditions of the one-stage ANAMMOX reactor. The nitrogen loading stage lasted for 71 days, and its purpose was to improve the nitrogen removal capacity of the one-stage ANAMMOX reactor, shorten the nitrogen removal time during the treatment of domestic sewage, and reduce the operational cost. The domestic sewage treatment stage lasted for 21 days. This approach was employed to determine the treatment effect and specific operating conditions of a one-stage ANAMMOX reactor to treat actual domestic sewage and provides a technical basis for the application of one-stage ANAMMOX reactors in domestic sewage treatment. To prevent DO from inhibiting ANAMMOX at the reactor startup period, the DO content of the regulating tank was controlled to be below 0.6 mg/L [21,28]. However, during the upward flow of wastewater, due to the presence of partial nitrification and other facultative anaerobic bacteria, the DO in the main reactor decreased in the vertical direction. The DO in the upper layer of the main reactor was below 0.1 mg/L.

During the start-up stage, the total nitrogen load rate (NLR) of the influent was set to 0.25 kg-N/m^3^/d. In the early stages of the experiment, the stability of the system was preliminarily determined by monitoring the effluent NH_4_^+^-N and NO_2_^−^-N contents. On the 27th day, after the removal of NH_4_^+^-N had stabilized, the operation of the system was assessed by increasing the effluent contents of NO_3_^−^-N and TN.

Following the start-up of the reactor, the influent TN concentration was maintained at approximately 200 mg/L. The NLR was increased by gradually reducing the HRT to determine the HRT and NLR that achieved the highest TN removal rate. The assessment of the nitrogen load-lifting stage was divided into five stages (Table 3). The HRT was gradually reduced from 15 to 4 h, and the NLR was gradually increased from 0.32 to 1.20 kg-N/m^3^/d.

To reduce the influence of COD on the system, the domestic sewage treatment experiment was divided into two stages, and the water distribution for each stage was 300 L. The contents of domestic wastewater and tap water during the first stage were 100 and 200 L, respectively, and an appropriate amount of NH_4_HCO_3_ was added to maintain the NH_4_^+^-N and TN concentration of the raw water. The contents of domestic wastewater and tap water in the second stage were 200 and 100 L, respectively. Similarly, an appropriate amount of NH_4_HCO_3_ was added to maintain the NH_4_^+^-N and TN concentrations of the raw water. Excluding the HRT, the other conditions remained the same as those during the start-up phase. The experiment was conducted for 21 days, with the first stage lasting seven days, and the HRT was 4 h. The second stage lasted 14 days, with HRTs of 4 h per day for the first four days and 6 h per day for the final 10 days.

### 2.2. Determination Methods

In this experiment, the temperature and pH were measured using a portable PHB-3 pH meter (Sanxin Instrument Factory, Shanghai, China), and the DO was measured using an HQ30d portable dissolved oxygen meter (HACH, USA). An online pH monitoring system was set up in the regulating tank (DPH10AC+DPH-SOC10, Tianjian Innovation Environment Technology Co., Ltd, Beijing, China). The routine test items included the COD, NH_4_^+^-N, NO_2_^−^-N, TN, and TP. The COD was determined following the standard method [29], the contents of NH_4_^+^-N and NO_2_^−^-N were determined by colorimetry following the standard method [29], and the TN content was determined via ultraviolet spectrophotometry with alkaline potassium persulfate digestion [29]. The TP content was determined following the method proposed by Yue et al. [30].

### 2.3. Microbiological Analytical Methods

The sludge sample used for microbiological analysis was obtained from the sludge produced after the nitrogen loading test. These samples were sequenced by Sangon Biotech (Shanghai) Co. Ltd., following the same method as Xiaoning et al. [27].

## 3. Results and Discussion

### 3.1. One-stage ANAMMOX Reactor Start-up

**Figure 3:**
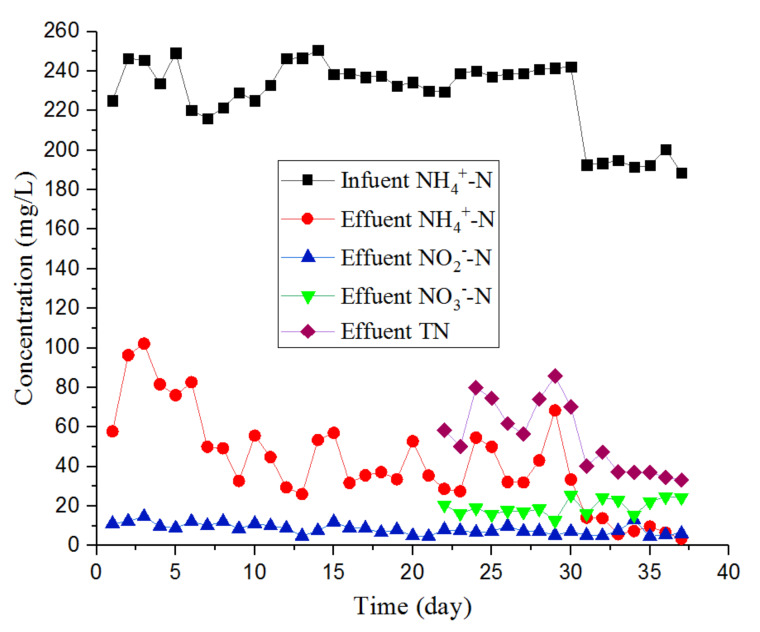
Changes in the contents of NH_4_^+^-N, NO_2_^−^-N, NO_3_^−^-N, and TN. Note: At the initial startup of the integrated ANAMMOX system.

The compositions of the artificial wastewater used in the start-up phase were the same as that of the laboratory-grown ANAMMOX bacteria. Therefore, the NH_4_^+^-N consumption of the one-stage ANAMMOX reactor in Figure 3 on the first 5 days remained at a relatively high level. From the 31st day to the end of the experiment, the NH_4_^+^-N content of the effluent of the system was stable, at approximately 10 mg/L, and the consumption of NH_4_^+^-N reached up to 98.1% (including the direct consumption of NH_4_^+^-N by microorganisms and the amount of NH_4_^+^-N converted to NO_2_^−^-N and NO_3_^−^-N). The inoculated and partial nitrifying sludge and ANAMMOX sludge were initially considered stable. The amount of NO_2_^−^-N in the effluent decreased, which also demonstrated that the stability of the ANAMMOX process in the system increased. After the system stabilized, the NO_2_^−^-N content of the effluent was maintained above 5 mg/L, indicating that the NO_2_^−^-N provided by partial nitrification was sufficient for ANAMMOX. The content of NO_3_^−^-N was higher than that of NO_2_^−^-N, with the former ranging from 12.7–25.6 mg/L. This is because 0.26 mol of NO_3_^−^-N was generated for every 1 mol of NH_4_^+^-N consumed during the ANAMMOX process. Meanwhile, a small amount of NO_3_^−^-N was likely generated during the aeration of the regulating tank. In this experiment, TN mainly originated from NH_4_^+^-N. In the final 16 days of the start-up phase, the TN content was between 50.2 and 85.8 mg/L during the first nine days, and the removal effect of TN was not stable, which may have been due to the high concentration of TN in the influent water. In the final seven days of the start-up phase, when the influent TN content decreased from approximately 250 to 200 mg/L, the TN removal rate exceeded 80%, indicating that the one-stage ANAMMOX reactor had started successfully. According to the research results of Hui et al. [31], a mixture of denitrifying sludge and ANAMMOX granular sludge can allow the start-up of the ANAMMOX process to complete within 40 days. In this study, the mixture of partial nitrifying and ANAMMOX granular sludge mixing also achieved this, indicating that ANAMMOX granular sludge and other types of activated sludge related to N conversion in the sewage mixing can start the ANAMMOX process more easily.

### 3.2. Nitrogen Load Acclimation Stage

**Figure 4:**
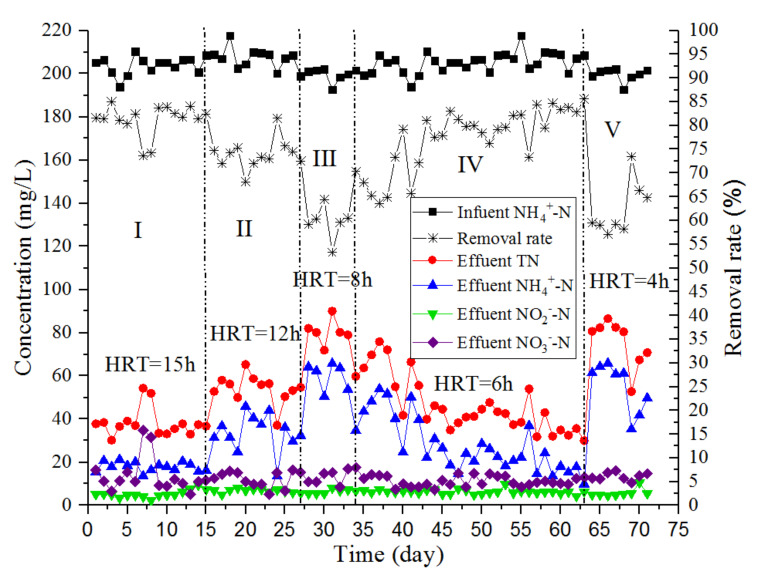
Changes in the contents of TN, NH_4_^+^-N, NO_2_^−^-N, and NO_3_^−^-N in the nitrogen-loading experiment.

During the high nitrogen load-acclimation experiment, the TN concentration of the influent was maintained at approximately 200 mg/L. The removal of TN, NH_4_^+^-N, NO_2_-N, and NO_3_^−^-N by the reactor is shown in Figure 4. The changes in the concentrations of NO_2_^−^-N and NO_3_^−^-N in water were lower than those of NH_4_^+^-N. The fluctuations in the concentration of NH_4_^+^-N in the effluent were mainly attributed to the changes in HRT, which was also the main cause of the fluctuations in the TN concentration of the effluent. According to Figure 4, the content of NO_2_^−^-N was maintained between 5.0 and 7.0 mg/L, indicating that the NO_2_^−^-N provided by partial nitrification met the requirements of the ANAMMOX reaction process. The average TN removal rate during Stages I and II was 78.0%, and the highest TN removal rate was 85.0%. During the first eight days of Stages III and IV, the TN content of the effluent fluctuated between 41.8 and 90.0 mg/L, and the TN removal effect was not stable. During the final 21 days of Stage IV, the average and maximum removal rates of TN were 80.8% and 85.6%, respectively. The average TN removal rate of the V effluent was 62.2%; this effluent type had a short HRT and a high NLR, resulting in the poor effluent removal rate achieved by the reactor. According to these analysis results, when the HRT and NLR are 6 h and 0.8 kg-N/m^3^/d, respectively, the one-stage ANAMMOX reactor achieved the highest nitrogen removal rate. To ensure an average TN removal rate of over 80%, the highest NLR of the one-stage ANAMMOX process was 0.8 kg-N/m^3^/d, which was lower than that of the CANON process (NLR was 1.2–8.9 kg-N/m^3^/d) [3]. However, this NLR is sufficient for domestic sewage treatment.

### 3.3. One-stage ANAMMOX Reactor for Treating Real Domestic Sewage

**Figure 5:**
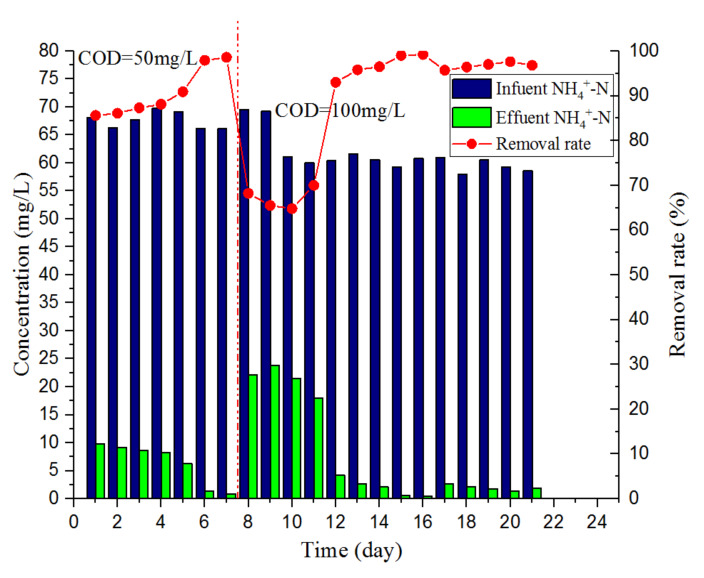
Changes in the content of NH_4_^+^-N.

Figure 5 shows the changes in the contents of NH_4_^+^-N between the influent and effluent water during the experiment. The influent NH_4_^+^-N content was approximately 60.0 mg/L. In the first stage, the COD of the influent was approximately 50 mg/L, the NH_4_^+^-N content of the effluent was maintained below 10.0 mg/L, and the highest NH_4_^+^-N removal rate was 98.6%. In the second stage, the COD of the influent was approximately 100 mg/L. During the first four days of this stage, the concentration of NH_4_^+^-N in the effluent increased rapidly (maximum NH_4_^+^-N content of the effluent was 23.8 mg/L), and the removal rate of NH_4_^+^-N decreased to 64.8%. According to the experimental results in Section 3.1 and Section 3.2, the consumption of NH_4_^+^-N in the one-stage ANAMMOX reactor mainly depended on partial nitrification and ANAMMOX. When the influent COD concentration was increased from 50 to 100 mg/L, the rapid proliferation of heterotrophic bacteria would inhibit the activity of ANAMMOX bacteria, resulting in an increase in the concentration of NH_4_^+^-N in the effluent. Through four days of environmental adaptation and extending HRT from 4 to 6 h, the NH_4_^+^-N content of the effluent was reduced from 23.8 to approximately 4.2 mg/L, and the NH_4_^+^-N removal rate recovered to 93.0%. In the second stage of stable operation, the NH_4_^+^-N content of the effluent was kept below 5.0 mg/L, and the average removal rate was 96.7%, demonstrating that the one-stage ANAMMOX reactor can effectively remove nitrogen during domestic sewage treatment.

**Figure 6:**
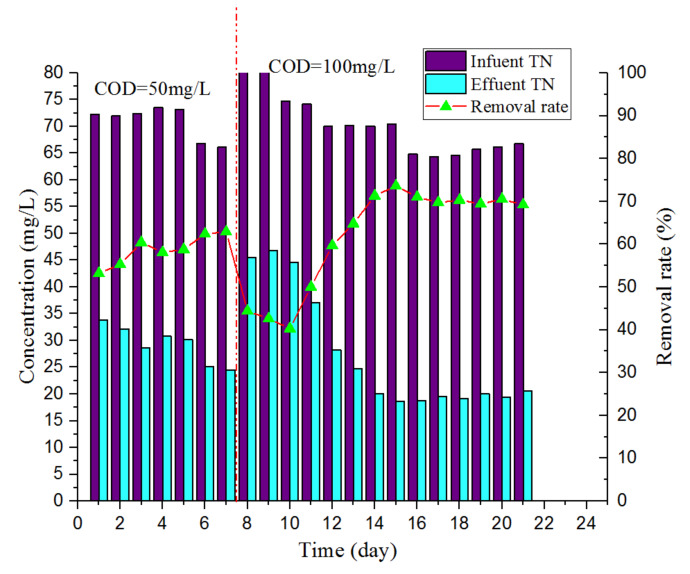
Changes in the content of TN.

Figure 6 shows the changes in the TN content of the influent and effluent water during the experiment. The influent TN was maintained at approximately 70.0 mg/L. When the COD of the influent water suddenly increased to 100 mg/L, the TN content of the effluent water fluctuated greatly during the first four days of the second stage, with the maximum TN concentration reaching 46.8 mg/L and the minimum TN removal rate reaching 44.4%. After a period of adaptation and upon extending the HRT from 4 to 6 h, the TN concentration in the effluent gradually decreased, and then finally remained at approximately 20.0 mg/L. The TN removal rate was approximately 70.0%.

**Figure 7:**
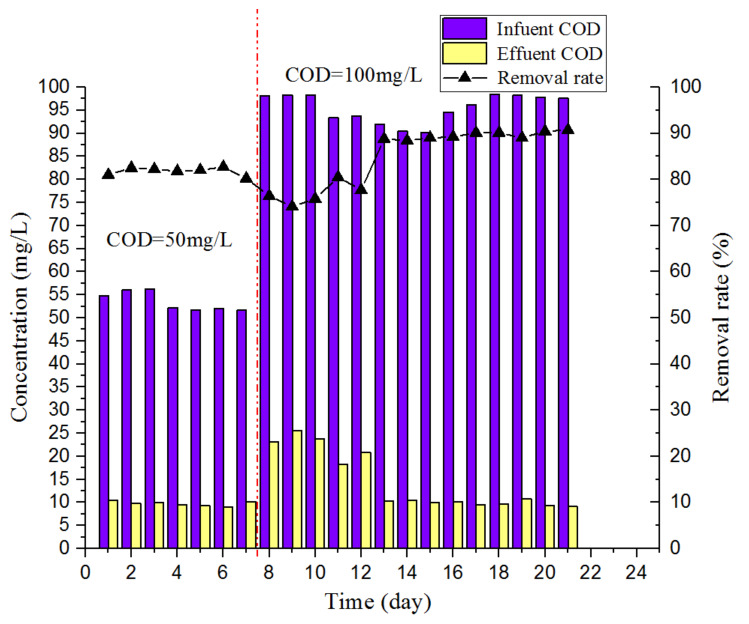
Changes in the COD.

Figure 7 shows the changes in the COD of the influent and effluent water during the experiment. In the first stage, the COD in the influent was approximately 50.0 mg/L, and the COD removal rate for the effluent was maintained above 80.0%. During the early period of the second stage, when the COD of the influent suddenly increased to approximately 100.0 mg/L, the COD of the effluent fluctuated significantly, and the minimum COD removal rate decreased to 74.1%. From the fifth day of the second stage, the HRT was extended from 4 to 6 h. After the reactor stabilized, the COD of the effluent was approximately 9.9 mg/L, and the COD removal rate remained at approximately 89.0%. According to the research results of Kato et al. [32] and Goel et al. [33], the supply of trace amounts of oxygen can accelerate the hydrolysis rate of enzymes in anaerobic digestion microorganisms and improve their activity. The DO in the main reactor was reduced from below 0.6 to below 0.1 mg/L from the bottom to the upper layer, so the anaerobic digestion microorganisms can still maintain a high activity in the middle and upper layers of the reactor, and the reactor can remove COD by anaerobic digestion. At the same time, the partial nitrification and denitrification processes in the reactor can also consume part of COD.

Too little DO will inhibit the activity of nitrifying bacteria. Therefore, to ensure that NH_4_^+^-N can be sufficiently oxidized, the DO of the nitrification tank during the traditional treatment of domestic sewage is generally maintained above 2.0 mg/L [34,35]. In this study, the average removal rates of the effluent NH_4_^+^-N and TN during the domestic sewage treatment experiment reached 96.7% and 70.0%, respectively, in the stable operation period, indicating that the ANAMMOX process can effectively remove nitrogen from domestic sewage. As the DO in the reactor was controlled to be below 0.6 mg/L, the aeration required was 233.3% lower than that required in the traditional nitrification/denitrification process. Therefore, the one-stage ANAMMOX reactor could effectively reduce the cost of sewage nitrogen removal during the treatment of domestic sewage.

We also found that the one-stage ANAMMOX reactor used to treat campus domestic sewage was sensitive to changes in COD, but the pollutants in the wastewater were effectively removed after adaptation. When the influent COD increases from 50 to 100 mg/L, various types of heterotrophic bacteria in the reactor will accelerate the proliferation and metabolic rate due to the increase of COD, thus, causing a certain inhibitory effect on the activity of ANAMMOX bacteria, and reducing the removal effect of effluent NH_4_^+^-N and TN. After an adaptation period of approximately one week, the activity of the ANAMMOX bacteria was restored, the microbial structure in the one-stage ANAMMOX reactor was restored to stability, and the system exhibited a good pollutant removal effect.

### 3.4. Microbial Diversity Analysis

To determine the microbial community of the one-stage ANAMMOX reactor after the start-up and nitrogen load domestication stages, sludge samples (T1, T2, and T3) collected after the load-up experiment were analyzed.

According to Table 4, there are 244 operational taxonomic units (OTUs) between T1 and T2, 247 OTUs between T1 and T3, 269 OTUs between T2 and T3, and 194 OTUs between T1, T2, and T3, indicating high similarity in the microbial diversity of the samples. The maximum number of OTUs in T1, T2, and T3 was 440, the maximum Chaol index value was 595.18, and the maximum Shannon diversity index value was 2.15, which are well below the values reported by Kwon et al. [36]. This result indicates that, following the start-up and nitrogen load stages of domestication, the microbial diversity in the one-stage ANAMMOX reactor was significantly reduced due to its unique operating conditions. The coverage indices of T1, T2, and T3 all exceeded 0.99, indicating that the sequencing depth mostly covered all sequences in the sample and that the sequencing results were valid and reliable. With the continuous operation of the reactor and strict control of the operating conditions required by the functional bacteria, the bacteria that were not suitable for the inorganic, dark, and anaerobic environments of the system were constantly eliminated; thus, the microbial diversity and abundance of the system were lower than those of the inoculated sludge during the start-up period. In an activated sludge ecosystem, if the primary functional bacteria exist and accumulate in the system, the stability of the activated sludge ecosystem is unrelated to changes in microbial diversity, and the stability of the system mainly depends on the growth and changes of the functional bacteria in the system.

**Figure 8:**
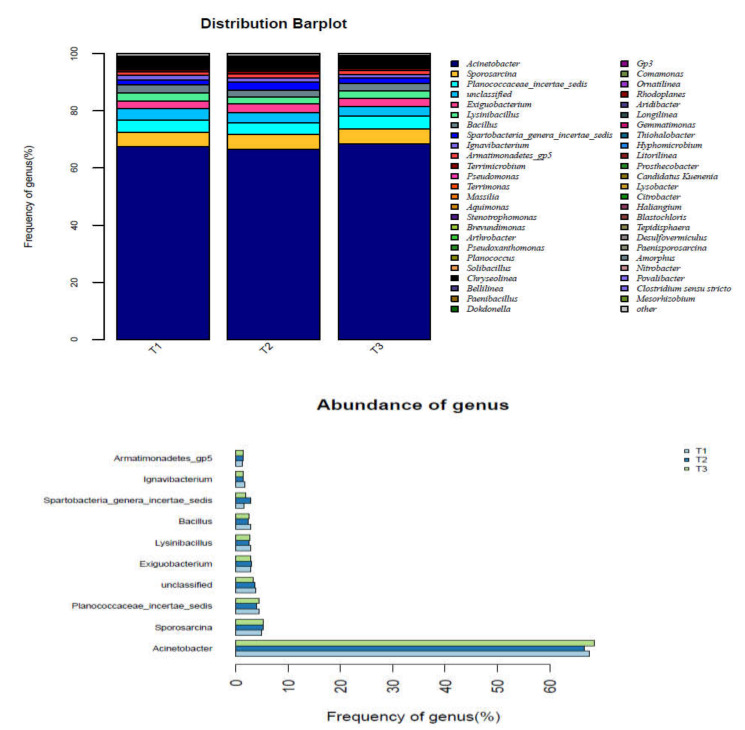
Distribution of microbial community structure.

To explore the types of bacteria in the reactor that were enriched and the changes in their corresponding abundance values, the sludge samples were compared and analyzed at the genus level, and the data processing results are presented in Figure 8. The diversity of the microbial community was rich; however, the distribution was not uniform, with *Acinetobacter* and *Sporosarcina* accounting for 67.53% and 5.12% of the total microbial content, respectively. *Acinetobacter* is a functional microorganism that consumes nitrogen in the reactor and is the same microorganism with strong nitrogen removal performance that was isolated from petrochemical wastewater in the treatment process by Lang et al. [37]. *Sporosarcina* mainly forms carbonate precipitates from minerals in wastewater [38]. Many *Proteobacteria* were enriched, and *Acinetobacter* became the dominant type of bacteria in the system. This is the result of the screening of the reactor under the inorganic, anaerobic, and dark operating conditions. Therefore, *Acinetobacter* are highly compatible with the one-stage ANAMMOX system, which can improve the efficiency of the system highly and achieve stable nitrogen removal performance.

There are many species of ANAMMOX bacteria, mainly belonging to the genus of *Brocadia*, *Kuenenia*, *Jettenia*, *Anammoxoglobus*, *Scalindua,* and *Anammoximicrobium*. Therefore, for different wastewater quality, process, and seed sludge, it was very likely that the dominant ANAMMOX bacteria strain in the process would be different. Through 16S RNA technology analysis, Strous et al. [39] found *Candidatus Brocadia* in the SBR of Delft laboratory, the Netherlands, which used NH_4_^+^-N and organic matter as the reaction matrix. Kartal et al. [40] found *Candidatus Brocadia fulgida* in the Rotterdam sewage treatment plant, the Netherlands, and the reaction matrix was also NH_4_^+^-N and organic matter. As for the landfill leachate, Liu et al. [41] found that the main ANAMMOX bacteria were *Candidatus Scalindua wagneri* and *Candidatus Scalindua brodiae*. The above research results are different from the ANAMMOX bacteria found in this study.

In this study, the start-up of a one-stage ANAMMOX reactor was completed quickly within 40 days using a mixture of partial nitrifying and ANAMMOX sludges. Only 130 days elapsed from the start-up stage to the normal operation of the reactor, which is significantly less than the 3.5 years that elapsed from startup to the normal operation of ANAMMOX technology achieved by the Dokhaven sewage treatment plant in the Netherlands [42]. Here, a one-stage ANAMMOX process was used to treat domestic sewage. After the system ran stably, the COD content of the effluent was approximately 9.9 mg/L, and the COD removal rate was approximately 89%. The NH_4_^+^-N content of the effluent was maintained below 5.0 mg/L, and the average removal rate of NH_4_^+^-N was 96.7%. The TN of the effluent was approximately 20.0 mg/L, and the TN removal rate was approximately 70%.

The discharge limits of COD, NH_4_^+^-N, and TN in the first level-A standard of the Chinese Discharge Standard for Pollutants from Urban Sewage Treatment Plants (GB18918-2002) are 50, 5, and 15 mg/L, respectively. According to the laboratory test results, the influent TN of domestic sewage from the campus of Guilin University of Technology (Yanshan campus) was 5.0–10.0 mg/L higher than that of the domestic sewage treatment plant in Yanshan town. Therefore, for urban domestic sewage with a low influent TN concentration, a one-stage ANAMMOX reactor can be used to meet the standard discharge content. For domestic sewage with a high TN content, according to the data collected for the final seven days of the start-up stage, the average proportion of NO_3_^−^-N in the effluent TN of the one-stage ANAMMOX reactor after stable operation was 57.17%. When combining the one-stage ANAMMOX process with traditional denitrification (denitrification–ANAMMOX), part of the effluent from the one-stage ANAMMOX reactor flows back to the denitrification tank, and the remaining NO_3_^−^-N in the sewage can be removed by denitrification; thus, the TN in the effluent will be further reduced to reach the standard discharge rate.

The one-stage ANAMMOX process has a simple structure, simple operation procedure, and low operation cost, which are great advantages for China’s plans for developing a new domestic sewage treatment process that conserves more energy and is environmentally sustainable. As ANAMMOX bacteria require high temperatures to maintain sufficient activity, the one-stage ANAMMOX process can be applied for domestic sewage treatment in the southern coastal areas of China (such as Guangdong Province, Guangxi Province, and Hainan Province) and other tropical areas, which will greatly reduce the cost of domestic sewage treatment.

## 4. Conclusions

A one-stage ANAMMOX reactor can be started quickly within 40 days by mixing partial nitrifying sludge with ANAMMOX sludge. The *Accinetobacter* in the one-stage ANAMMOX reactor became the dominant strain of the system after nitrogen load acclimation, and the system exhibited high efficiency and a stable nitrogen removal performance. When the content of domestic sewage in the influent increased from 1/3 to 2/3, the one-stage ANAMMOX reactor was sensitive to changes in the COD during the treatment of campus domestic sewage. However, after a period of adaptation, the system effectively degraded the pollutants in the sewage.

## Figures and Tables

**Table 1:** Composition of Wastewater.

Parameter	Unit	Startup Stage	High Nitrogen Load Acclimation Stage	Domestic Sewage
Chemical oxygen demand (COD)	mg/L	--	--	140–160
NH_4_+-N	mg/L	250–200	200 ± 5	50–70
Total nitrogen (TN)	mg/L	250–200	200 ± 5	70–80
Total phosphorus (TP)	mg/L	--	--	3–4
KH_2_PO_4_	mg/L	25	25	--
NaHCO_3_	mg/L	1000	1000	--
CaCl_2_·2H_2_O	mg/L	113	113	--
MgSO_4_·7H_2_O	mg/L	100	100	--
pH	--	7.5–7.6	7.5–7.6	6.7–8.2
Temperature	°C	30 ± 1	30 ± 1	23.2–26.7

**Table 2:** Operating conditions of the reactor.

Stage	Duration (d)	HRT (h)	Temperature (°C)	pH	DO (mg /L)	Cycle Ratio
Start-up	1–37	24	30 ± 1	7.5–7.6	< 0.6	4:1
High nitrogen load acclimation	38–108	15-4	30 ± 1	7.5–7.6	< 0.6	4:1
Treatment of domestic sewage	109–129	4-6	30 ± 1	7.5–7.6	< 0.6	4:1

**Table 3:** Operating conditions of the reactor.

Phase	Duration (d)	NLR (kg-N/m^3^/d)	HRT (h)	Temperature (°C)	pH	DO (mg /L)	Cycle Ratio
I	1–15	0.32	15	30 ± 1	7.5–7.6	< 0.6	4:1
II	16–27	0.40	12	30 ± 1	7.5–7.6	< 0.6	4:1
III	28–34	0.60	8	30 ± 1	7.5–7.6	< 0.6	4:1
IV	35–63	0.80	6	30 ± 1	7.5–7.6	< 0.6	4:1
V	64–71	1.20	4	30 ± 1	7.5–7.6	< 0.6	4:1

**Table 4:** Correlation index of the alpha diversity of the sludge samples.

Sample	Quality Sequence	OTUs	Chaol	Shannon	Coverage
T1	34878	381	533.24	1.84	0.9960
T2	45936	440	595.18	2.15	0.9965
T3	44230	430	564.57	1.78	0.9965
